# How Halide Alloying Influences the Optoelectronic Quality in Tin-Halide Perovskite Solar Absorbers

**DOI:** 10.1021/acsenergylett.3c01241

**Published:** 2023-08-28

**Authors:** Felix J. Berger, Isabella Poli, Ece Aktas, Samuele Martani, Daniele Meggiolaro, Luca Gregori, Munirah D. Albaqami, Antonio Abate, Filippo De Angelis, Annamaria Petrozza

**Affiliations:** † Center for Nano Science and Technology @PoliMi, Istituto Italiano di Tecnologia, via Rubattino 81, 20134 Milano, Italy; ‡ Department of Chemical, Materials and Production Engineering, University of Naples Federico II, Piazzale Vincenzo Tecchio 80, 80125 Napoli, Italy; § Physics Department, Politecnico di Milano, Piazza L. da Vinci, 32, 20133 Milano, Italy; ∥ Computational Laboratory for Hybrid/Organic Photovoltaics (CLHYO), Istituto CNR di Scienze e Tecnologie Chimiche “Giulio Natta” (CNR-SCITEC), 06123 Perugia, Italy; ⊥ Department of Chemistry, Biology and Biotechnology, University of Perugia, 06123 Perugia, Italy; # SKKU Institute of Energy Science and Technology (SIEST) Sungkyunkwan University, Suwon 440-746, Republic of Korea; ∇ Chemistry Department, College of Science, King Saud University, Riyadh 11451, Saudi Arabia

## Abstract

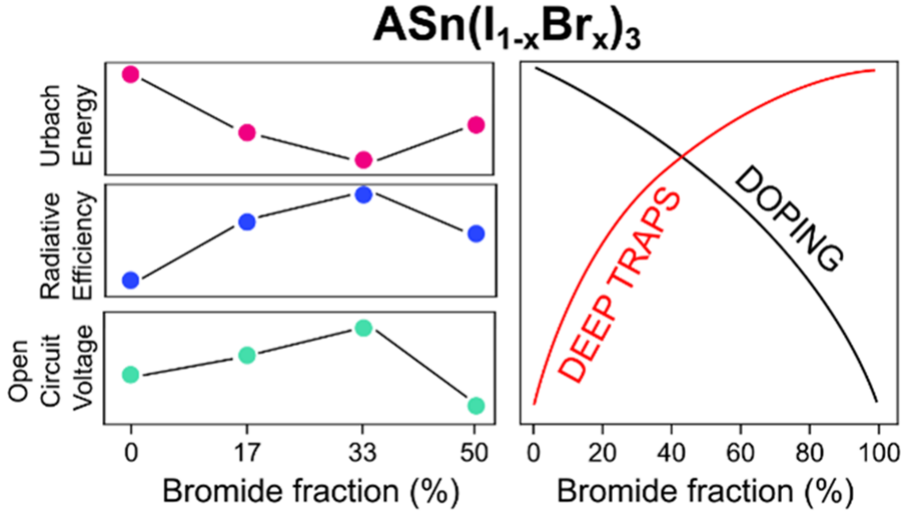

Halide alloying in tin-based perovskites allows for photostable bandgap tuning between 1.3 and 2.2 eV. Here, we elucidate how the band edge energetics and associated defect activity impact the optoelectronic properties of this class of materials. We find that by increasing the bromide:iodide ratio, a simultaneous destabilization of acceptor defects (tin vacancies and iodine interstitials) and stabilization of donor defects (iodine vacancies and tin interstitials) occurs, with strong changes arising for Br contents exceeding 50%. This translates into a decreased doping which is, however, accompanied by a higher density of nonradiative recombination channels. Films with high Br content show a high degree of disorder and trap state densities, with the best optoelectronic quality being found for Br contents of around 33%. These observations match the open circuit voltage trend of tin-based mixed halide perovskite solar cells, supporting the relevance of optoelectronic properties and chemistry of defects to optimize wide-bandgap tin perovskite devices.

Tin-based metal halide perovskites have emerged as highly promising materials for efficient single-junction solar cells^1−3^ and silicon-perovskite or all-perovskite tandem photovoltaics,^4−8^ exploiting the possibility of tuning their bandgap.^9^ To date, however, this possibility has been mainly explored by engineering the metal cation composition, mixing lead with tin.^10^ It is well accepted that pure tin-based perovskites have a defect chemistry mainly dictated by Sn defects,^11,12^ such as the easy formation of tin vacancies, which are shallow traps close to the valence band and therefore introduce a large density of background holes (p-doping).^13−15^ These same defects, along with the low tin oxidation potential compared to that of lead, are also related to the facile oxidation of tin(II) to tin(IV).

Very recently, we demonstrated that mixed-halide Sn perovskites do not show the notorious bandgap photoinstability under photoexcitation typical of their lead counterpart, which is related to the formation of I-rich phases within the thin films.^16^ To date, only a few reports have shown the use of mixed halide I–Br tin perovskite thin films to fabricate wider bandgap semiconductors and larger open circuit voltage solar cells.^17−19^ In this work, we provide a comprehensive picture of the radiative and nonradiative recombination processes in MASn(Br_*x*_I_1–*x*_)_3_ thin films, as a function of the halide composition, tuned from pure iodide (*x* = 0) to pure bromide (*x* = 1). By combining photoluminescence measurements and density functional theory (DFT) calculations, we show that halide alloying in MASn(Br_*x*_I_1–*x*_)_3_ offers a way to modulate doping in tin perovskite films by modifying the density of acceptor/donor defects and to simultaneously affect the probability of having charge trapping states which cause carrier losses. Eventually, we demonstrate that the optoelectronic properties of Sn mixed halide perovskite thin films, observed as a function of the Br:I ratio, directly mirror the performance of tin-based mixed halide perovskite solar cells, with the highest open circuit voltage measured for cells with 33% of Br. This composition appears indeed to represent a balance between thin-film doping and the appearance of nonradiative charge carrier traps, opening the way to stoichiometrically engineered tin-based perovskites.

We prepared a series of thin films of ASn(Br_*x*_I_1–*x*_)_3_ (where A = MA (methylammonium) or FA (formamidinium)) in which the halide composition is tuned from pure iodide (*x* = 0) to pure bromide (*x* = 1). The incorporation of bromide within the structure is confirmed by the X-ray diffraction (XRD) patterns shown in Figures S1 and S2. As the bromide concentration increases, a gradual shift of the XRD peaks occurs. This phenomenon is consistent with a gradual contraction of the unit cell volume due to the smaller ion radius of bromide compared to iodide. The inclusion of Br within the perovskite lattice is also confirmed by the shift of the photoluminescence spectra to higher energies (Figures S3 and S4). The top-view scanning electron microscopy (SEM) images of mixed halide thin films with different I:Br ratios show that grains slightly enlarge with Br incorporation (Figures S5 and S6), especially for FA compositions, while the thickness of the films is about 200 nm and does not change with Br content (Figure S7).

From now on, we will focus on MA-based materials being the archetype perovskite and the model system that received the most intense investigation so far, without the use of any additive, e.g. SnF_2_. The absorption edge of the material blue shifts with increasing Br fraction (Figure 1a), and bandgaps that range between 1.3 and 2.2 eV are obtained (Figure S8). Only the pure iodide MASnI_3_ film (*x* = 0) does not follow this trend and displays a blue-shifted absorption spectrum without a clear band onset, which might be due to its high doping level and concomitant Burstein–Moss effect.^15^ Interestingly, the photoluminescence (PL) spectra in Figure 1b show strong changes in the emission efficiency of the materials. It should be emphasized that these PL spectra were normalized with respect to the optical density extracted from Figure 1a. We further accurately measured the absolute photoluminescence quantum yield (PLQY) of thin films using an integrating sphere and observed the same trend with PLQY values that peak in MASn(I_1–*x*_Br_*x*_)_3_ with *x* = 0.33 (Figure S9). The PL efficiency of halide perovskites is extremely sensitive to both doping and trap defect densities.^14^ More specifically, we know that the presence of doping introduces a radiative pseudomonomolecular decay component,^15,20^ which enhances the PL efficiency, while traps contribute to a nonradiative decay component that reduces the PL efficiency.^21^ As the Br fraction increases, the PL intensity passes through a maximum at around *x* = 0.33, then drops and even becomes undetectable for the pure bromide composition (*x* = 1). The PL emission of MASnBr_3_ can be measured only when the film is excited with densities higher than 200 W cm^–2^ (Figure S10). Such a nonmonotonic trend in PL intensity points toward a complex interplay between doping and trap density as a function of halide composition that governs the optoelectronic properties of mixed-halide THPs.

**Figure 1:**
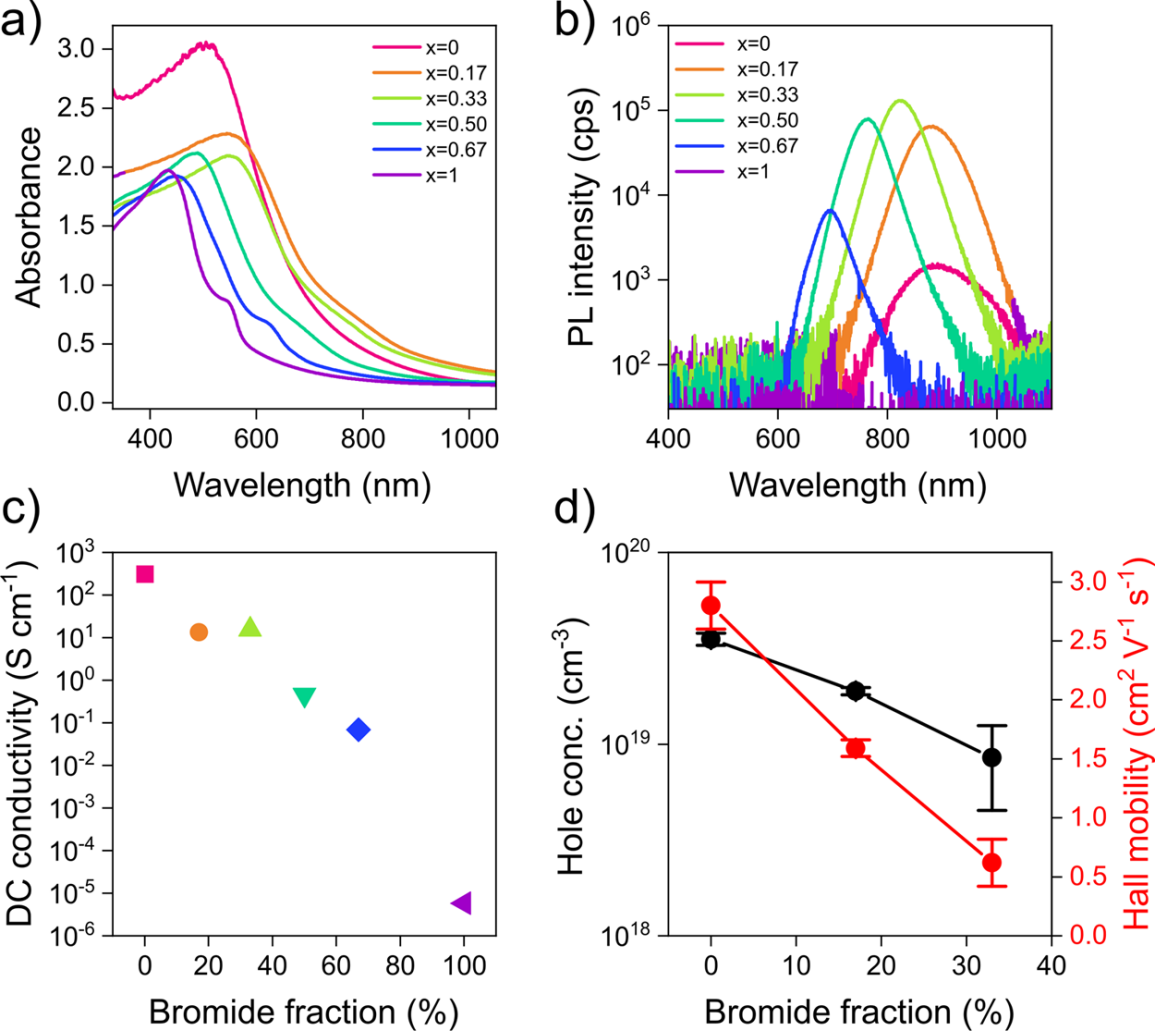
(a) UV–vis–NIR absorption of MASn(Br_*x*_I_1–*x*_)_3_. (b) PL spectra of MASn(Br_*x*_I_1–*x*_)_3_ thin films, normalized with respect to the optical density (excitation wavelength 450 nm, ∼100 mW cm^–2^). (c) DC conductivity of MASn(Br_*x*_I_1–*x*_)_3_ thin films measured by the two-point probe method. (d) Doping concentration and Hall mobility in MASn(Br_*x*_I_1–*x*_)_3_ thin films extracted from Hall effect measurements.

Conductivity measurements indicate a dramatic drop of conductivity by 8 orders of magnitude as the halide is varied from iodide to bromide (Figure 1c). This reduction in conductivity may arise from (i) the suppression of self-doping, (ii) a drop in carrier mobility, or (iii) a combination of both. To disentangle these contributions, Hall effect measurements were performed (Figure 1d). Due to the strong nonlinear increase in resistivity with bromide fraction (Figure S11), which increases by 2 orders of magnitude going from *x* = 0.33 to *x* = 0.5 compositions, our DC Hall effect setup provided reliable data only up to *x* = 0.33. The largest deviation in resistivity has been observed for MASn(I_1–*x*_Br_*x*_)_3_ films with *x* ≥ 0.5 where undesired side effects obscured the small Hall voltage. Both the level of p-doping and the Hall mobility decrease substantially upon admixing of bromide, from *p* = 4 × 10^19^ cm^–3^ and μ_H_ = 2.8 cm^2^ (V s)^−1^ for *x* = 0 down to *p* = 9 × 10^18^ cm^–3^ and μ_H_ = 0.6 cm^2^ (V s)^−1^ for *x* = 0.33 (see Table S1 for Hall effect parameters). Simultaneously, however, the PL intensity increases, as shown in Figure 1b. At first glance this may appear counterintuitive, as the bright emission of THPs is usually attributed to pseudomonomolecular radiative recombination of electrons with background holes and should therefore decrease upon dedoping. However, we recently demonstrated that the strong self-doping of iodide-based THPs can push the background hole density into a range (>10^19^ cm^–3^) in which the radiative efficiency becomes limited by Auger recombination even for low pump fluences.^14^ Indeed, fluence-dependent PL measurements confirmed that this picture holds also for the mixed-halide THPs investigated here (Figure S12). Hence, the brightening of PL upon admixing of bromide may be interpreted as a slowing down of Auger recombination due to dedoping. We know that one of the most common additives used in the field to reduce the background carrier concentration is SnF_2_. Therefore, we also checked the conductivity trend on mixed alloyed Sn samples fabricated with addition of 10 mol % SnF_2_ to check whether the halide alloying could deliver similar results. Figure S13 shows that the conductivity of films fabricated with SnF_2_ is overall lower than those measured on pristine samples, suggesting a reduced p-doping due to destabilization of Sn vacancy defects, and that the trend with Br addition is confirmed.

Thus, halide alloying offers a way to suppress self-doping in THPs and thereby optimize their radiative efficiency and potentially carrier diffusion lengths for device applications. On the other hand, the concomitant drop in carrier mobility points toward the formation of defects that act as trapping and/or scattering sites impeding charge transport, which eventually might be the main cause for the decrease of PL efficiency at higher bromide fractions.

A complementary tool to probe defects and doping in perovskites is photothermal deflection spectroscopy (PDS). The obtained absorption spectra (Figure 2a) feature a sharp drop at the band edge along with a broad tail due to free-carrier absorption (FCA) characteristic of doped perovskites. Commonly, the steepness of the absorption onset is related to the degree of disorder in the semiconductor and may be quantified by the Urbach energy extracted from a monoexponential fit to the band edge.^22^Figure 2b shows the integrated FCA signal and Urbach energy as a function of bromide fraction. The consistent decrease of the FCA with bromide content suggests that the admixing of bromide dedopes the semiconductor across the full compositional range and thus contributes to the observed loss of conductivity (compare Figure 1c). The Urbach energy, on the other hand, drops from a relatively large value of ∼60 meV for the pure iodide to a minimum of ∼33 meV around *x* = 0.33, thus indicating improved material quality at moderate bromide fractions in agreement with bright PL from such compositions. Beyond *x* = 0.33, the Urbach energy gradually increases and reaches a maximum value of ∼70 meV for the pure bromide phase, revealing a high degree of disorder. In agreement with that, the absorption spectrum for *x* = 1 features an unusually strong sub-bandgap absorption roughly 400 meV below the band edge. Most likely, this deep trap state is responsible for quenching the PL of the pure bromide THP (Figure 1b). In summary, the steady-state spectroscopic data suggest that the radiative efficiency of iodide-rich THPs is limited by self-doping and Auger recombination, whereas high trap densities and low doping levels prevail in bromine-rich compositions.

**Figure 2:**
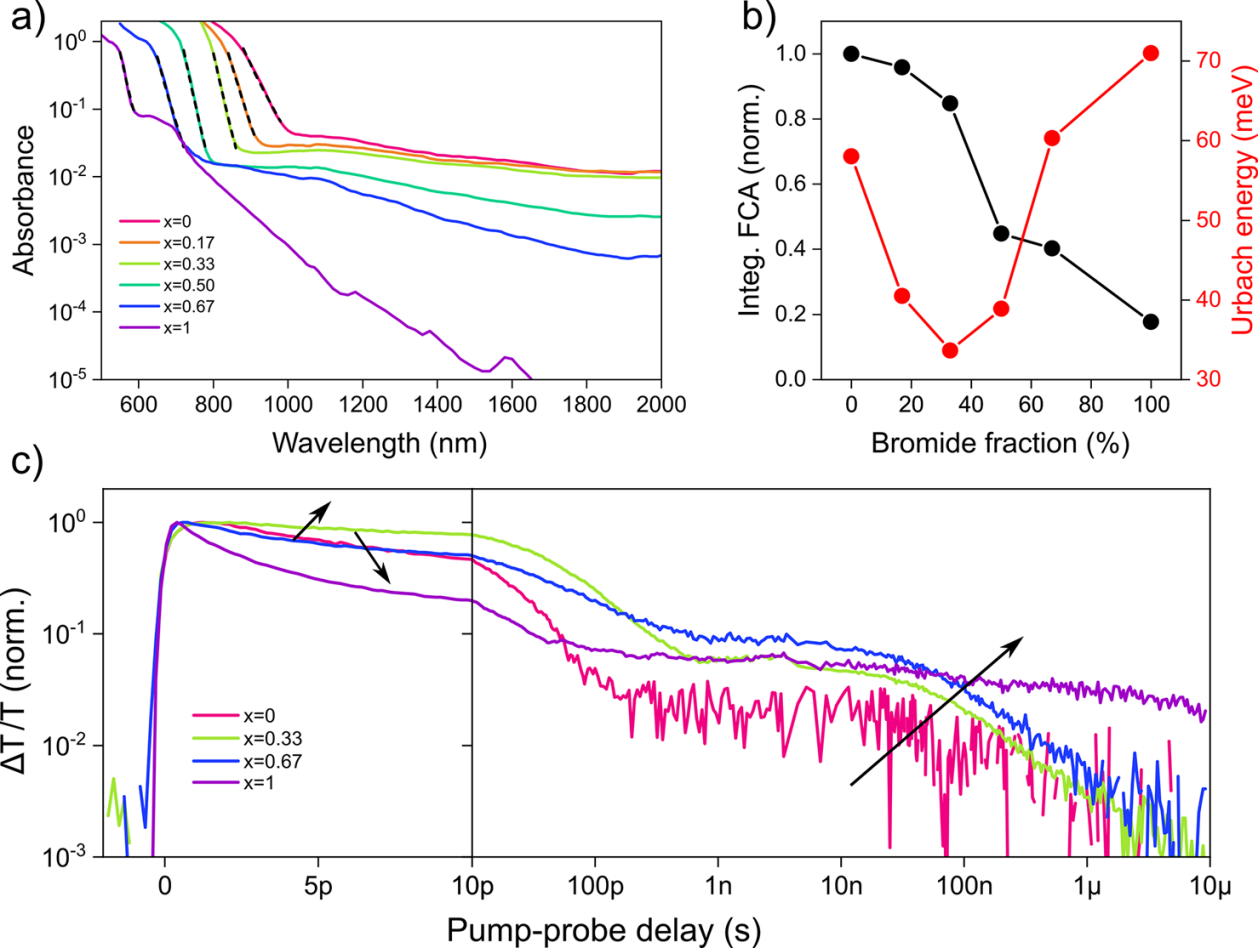
(a) Absorption spectra measured with photothermal deflection spectroscopy along with monoexponential fits to the absorption edge. (b) Spectrally integrated free-carrier absorption signal and Urbach energy as a function of bromide fraction. (c) Normalized kinetics at the band edge photobleach obtained from transient absorption spectroscopy. The arrows indicate increasing bromide fraction.

To further support the trends outlined above and to better understand the nature of the involved trap states, transient absorption (TA) spectroscopy was employed. The TA spectra are dominated by two photobleach signals (Figures S14 and S15), one centered at the band edge and one higher in energy, in analogy to the behavior observed for lead-based perovskites.^23^ We find similar lifetimes for both transients, in agreement with the commonly assumed picture of optical transitions between a split valence band and a shared conduction band. In the following, we focus on the dynamics of the band-edge photobleach as a probe of the density of photocarriers at a given time delay after the pump. Figure 2c shows the bleach dynamics for four characteristic halide compositions (data for all samples are given in Figure S16). All samples feature an initial fast decay on the order of ≤100 ps due to Auger decay, radiative recombination, and trapping, followed by a tail that stretches beyond 1 μs resulting from the presence of long-lived trapped carriers slowing down the recombination of their free counterparts. While in an intrinsic semiconductor either electron or hole traps could be responsible for this tail,^24^ in p-doped semiconductors, such as iodine-rich THPs, only electron traps (leaving behind free holes) can give rise to such a behavior, as free electrons could always recombine with background holes.

Upon moving from the pure iodide to *x* = 0.33, the initial decay is slowed down, which may be understood in terms of dedoping and reduced Auger recombination, in agreement with the improved PL efficiency. At larger bromide fractions, however, the decay is substantially sped up and reaches a time scale of a few picoseconds for the pure bromide. This can be attributed to fast carrier trapping that eventually dominates over radiative and Auger recombination due to increasing defect densities and progressive dedoping. At the same time, the long-lived tail consistently increases with the bromide content, pointing toward the creation of additional traps. For the pure bromide, trapping is very fast and the trapped carriers become extremely long-lived (≥10^–5^s ), thus rationalizing the negligible PL from this material.

To investigate the factors determining the different photophysics of perovskite materials at different bromide contents, we studied the defects chemistry of the MASnI_1.5_Br_1.5_ and MASnBr_3_ phases, by carrying out a comparison with the prototypical MASnI_3_ full tin perovskite. DFT calculations in the supercell approach have been performed to estimate the densities of the most common halide and metal defects in these materials, to understand their trapping activity, as well as their impact on the intrinsic carrier density. Due to the predominance of acceptor (valence band related) defects in tin-based perovskites,^25^ DFT calculations have been performed by using the hybrid PBE0 functional^26,27^ but neglecting spin–orbit coupling (SOC) effects, which mainly affects the conduction band (CB) energetics. The latter are, however, incorporated to obtain accurate material band gaps (see Computational Details in the Supporting Information). By moving from pure iodide to pure bromide perovskites, band gaps of 1.37, 1.95, and 2.14 eV are predicted for the MASnI_3_, MASnI_1.5_Br_1.5_, and MASnBr_3_ phases, respectively. The trend in the band gap variation versus the Br content is not linear, consistent with experimental values.

The band gap widening associated with the increase in the Br content has a remarkable impact on the stability of defects in the different phases, particularly for low Br contents. In Figure 3 the defect formation energies (DFE) and thermodynamic ionization levels (TIL) diagrams of the most common halide and tin defects in the three phases are reported. As previously discussed in the literature,^12^ the heavy p-doping of MASnI_3_ is ascribed to the high stability of tin vacancies (V_Sn_) and iodide interstitial (I_i_) acceptor defects, which pin the Fermi level to the top of the VB with estimated hole densities of ∼10^19^ cm^–3^. Notably, the densities of donor defects, i.e., V_I_ and Sn_i_, at the native Fermi level is on the order of 10^9^ cm^–3^, much lower than for acceptor defects. V_I_ and Sn_i_ defects show deep levels in the band gaps associated with electron trapping on undercoordinated tins.^12^

**Figure 3:**
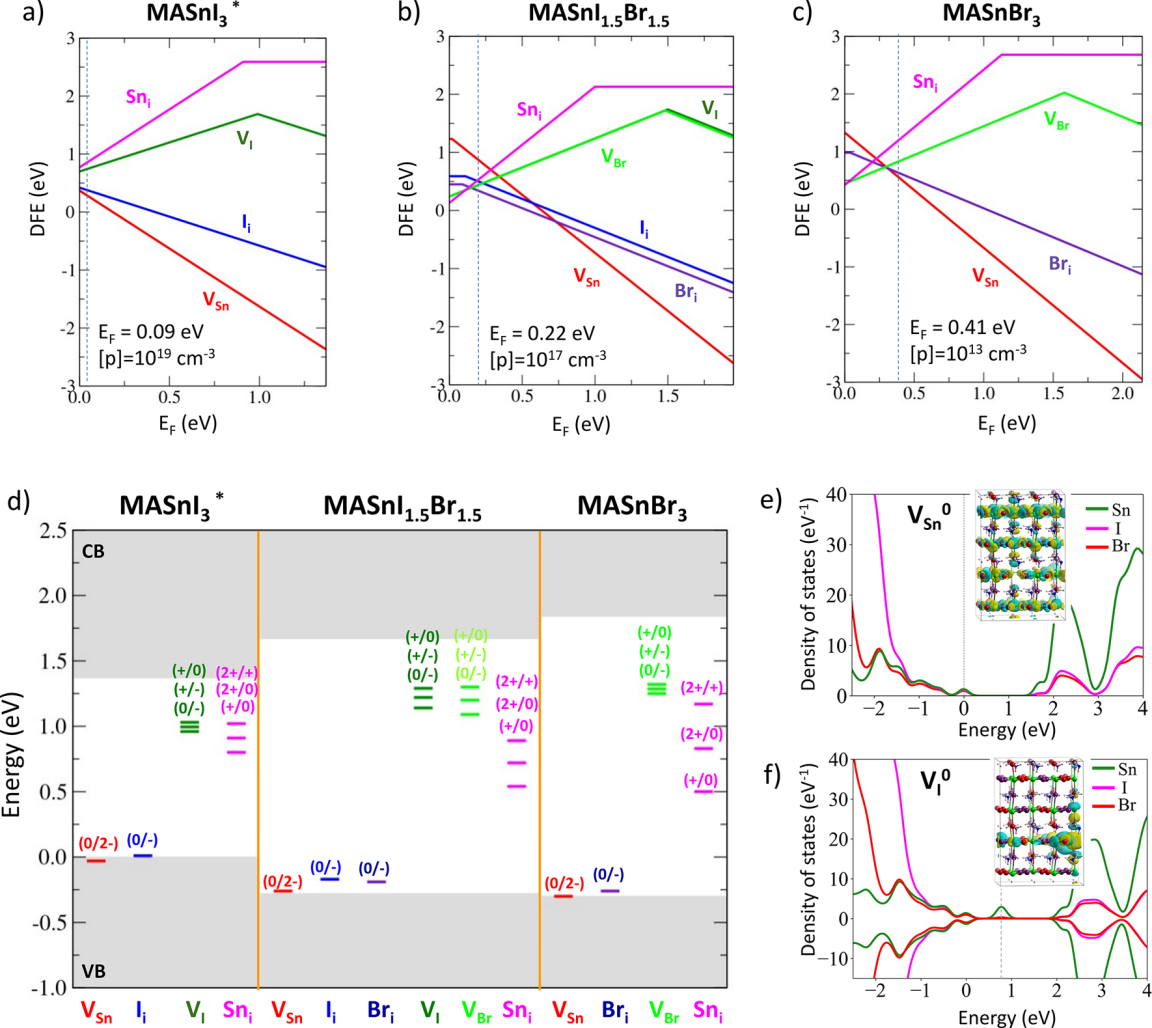
Defect formation energies calculated in halide-medium conditions of growths of (a) MASnI_3_, (b) MASnI_1.5_Br_1.5_, and (c) MASnBr_3_. (d) Thermodynamic ionization levels (TIL) of defects in the three phases. (e, f) Projected density of states over the atomic orbitals (pdos) and orbital isovalue plots of the V_Sn_^0^ and V_I_^0^ defects in MASnI_1.5_Br_1.5_, highlighting the different nature of the defects (shallow vs deep). For V_Sn_^0^ the orbital plot represents the lowest unoccupied molecular orbital (LUMO) associated with the delocalized hole, while in the case of the V_I_ the highest occupied state (HOMO) is reported, showing the electron localization on undercoordinated tin. The asterisks denote that defect quantities for MASnI_3_ have been evaluated at the experimental cell, whose parameters slightly differ from those obtained by the DFT optimization procedure.

By introduction of Br to form the mixed MASnI_1.5_Br_1.5_ phase, destabilizations of tin vacancies and iodide interstitials of ∼0.7 and ∼0.2 eV are observed, respectively, with a parallel stabilization of the V_I_ and Sn_i_ donor defects. In line with the nonlinear trend predicted by DFT for the band gaps of the materials, DFE changes are less pronounced for Br contents beyond 50%. DFE changes are readily understood by looking at the band edge modulation due to the halide alloying. The wider band gaps associated with high Br contents are accompanied by a lowering of the VBM energy compared to the full iodide perovskite, i.e. the VBM is more negative with respect to the vacuum level, thus destabilizing the acceptor defects. Notably, in the mixed MASnI_1.5_Br_1.5_ phase, bromide interstitials are more stable than iodide interstitials, while the halide vacancies show a comparable stability. The higher stability of Br_i_^–^ with respect to I_i_^–^ mostly lies in the reduced ionic radius and higher electronegativity of the Br ion compared to I.

The destabilization (stabilization) of acceptor (donor) defects moves the equilibrium Fermi level to values of 0.2 and 0.4 eV above the VBM for MASnI_1.5_Br_1.5_ and MASnBr_3_, respectively, indicating that halide alloying is a suitable strategy to reduce the p-doping of the tin perovskites. Most notably, the predicted reduction of the hole density based on our calculations is by up to 6 orders of magnitude compared to the full tin MASnI_3_ perovskite (see Figure 3a–c). Interestingly, the defects chemistry of the alloyed phases is dominated by halide Frenkel couples, i.e., V_Br/I_ and (Br/I)_i_, which are the most stable defects at the native Fermi level.

Although Br mixing is able to dedope the tin perovskite by modifying the density of acceptor/donor defects, it does not alter their charge trapping behavior. As can be seen in Figure 3d, tin vacancies and halide interstitials keep their original shallow nature by showing ionization levels resonant with the VB and no charge carrier localization in any case (see also Figure 3e). On the other hand, halide vacancies and tin interstitials show deep levels in the band gaps of the materials associated with the trapping of electrons from the CB (see Figure 3f). The highest activity of halide vacancies in the alloyed phases may thus activate nonradiative recombination channels and be responsible for the reduced PL efficiency at high Br ratios. For low Br ratios, as in the MASnI_2_Br phase, DFT analysis predicts lower densities of halide vacancies (higher formation energies) with respect to MASnI_1.5_Br_1.5_, while shallow acceptor defects are sensibly destabilized compared to the full iodide perovskite (Table S2 in the Supporting Information). Therefore, halide alloying may be employed to enhance the optoelectronic quality of THPs within the window from *x* = 0.17 to 0.5.

However, it should be noted that while an increase in the density of V_Br/I_ is predicted moving from MASnI_3_ to MASnI_1.5_Br_1.5_ (10^9^ vs 10^14^ cm^–3^), a progressive decrease is expected when moving to MASnBr_3_ (down to 10^9^ cm^–3^) due to the higher energy required for the formation of the respective Frenkel couple (0.8 eV in MASnI_1.5_Br_1.5_ and 1.5 eV in MASnBr_3_). Hence, the poor PL properties of MASnBr_3_ cannot be explained by the defect activity alone, but they can have multiple origins. Recently, Ouhbi et al. predicted that THPs, especially those with lighter halides (Br, Cl), support the formation of a small electron bipolaron state, which is typically characterized by very low carrier mobility and nonradiative decay.^28^ This, however, requires the concomitant trapping of two electrons at the same site; therefore, it could become relevant under high fluences. To gather more evidence in favor of small electron bipolaron state or defects acting as trap states, we measured the TA as a function of the pump fluence. Ideally, a reduction of the long-lived tail of the TA signal with pump fluence would hint at a trap state filling, while an increase of the long-lived tail with power would suggest the presence of bipolaronic states whose formation is indeed favored at high excitation densities. Figure S17 in the Supporting Information shows that the long-lived tail increases with pump fluence for all Br-containing perovskites, while it does not considerably change in pure iodide thin films, possibly supporting the presence of a small electron bipolaron state in mixed halide samples, as predicted by Ouhbi et al. A combination of defects and small bipolaronic states might thus explain the much reduced photoluminescence emission intensity observed in MASnBr_3_.

Finally, we explored the impact of halide alloying on the performance of DMSO-free perovskite solar cells in the state-of-the-art device architecture with ITO/PEDOT/Al_2_O_3_/FASn(I_1–*x*_Br_*x*_)_3_/C60/BCP/Ag sandwich architecture, with *x* = 0, 0.17, 0.33, and 0.5.^29^Figure 4a shows the current density–voltage (*J*–*V*) curves of FASn(I_1–*x*_Br_*x*_)_3_ mixed halide perovskites with different Br:I ratios measured under simulated AM 1.5G illumination (the main photovoltaic parameters are listed in Table S3). As expected, the short circuit current decreases with Br content due to widening of the bandgap (incident photon to current efficiency, IPCE, and integrated current are shown in Figure S18). Furthermore, a consistent increase of the open circuit voltage would be expected as the Br content increases due to the larger bandgap. In contrast to that, we observe a gradual increase of the open circuit voltage only up to a 33% content of Br, while it is drastically reduced in FASnI_1.5_Br_1.5_ solar cells (Figure 4b), in line with the optical and electronic characterization of the semiconductor thin films and computational calculation predictions.

**Figure 4:**
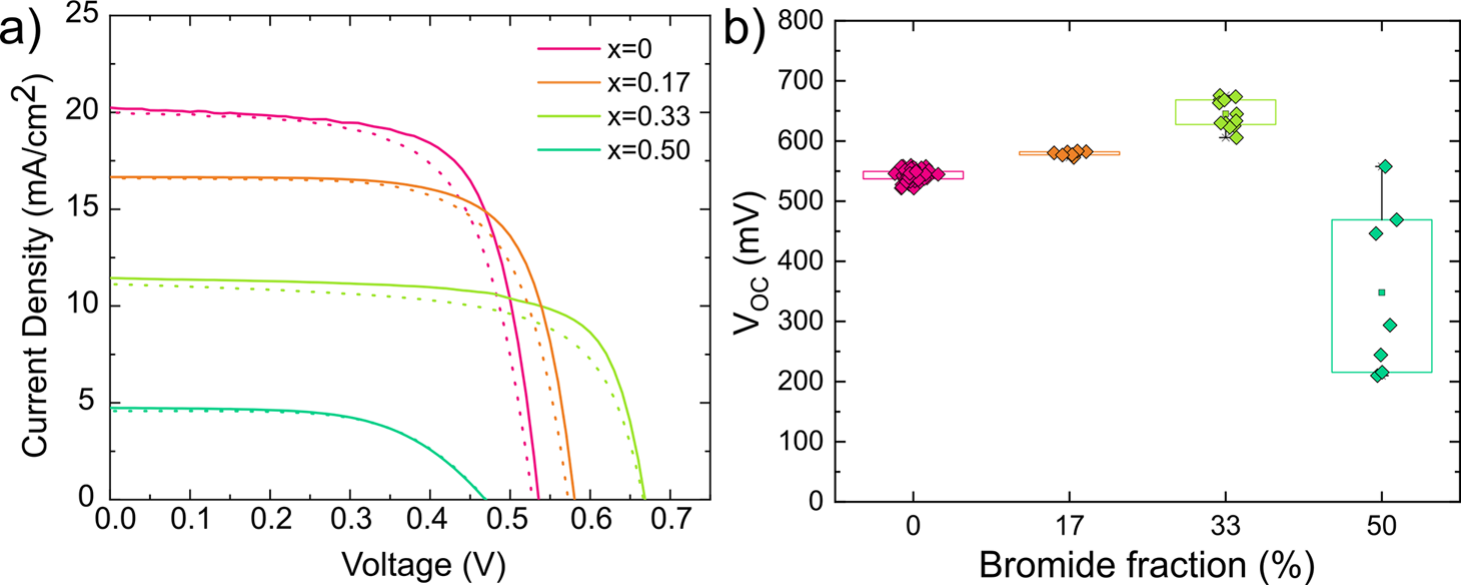
(a) *J*–*V* curves of FASn(I_1–*x*_Br_*x*_)_3_ solar cells with *x* = 0, 0.17, 0.33, and 0.5. The solid curve is the *J*–*V* curve measured under forward scan, while the dotted line indicates the *J*–*V* curve measured under reverse scan. (b) Box plots of the open circuit voltage of FASn(I_1–*x*_Br_*x*_)_3_ solar cells with *x* = 0, 0.17, 0.33, and 0.5, measured under forward scan.

In conclusion, we rationalize the role of defects on the optoelectronic properties of tin based perovskites as a function of the halide composition. The modulation of the energy edges acts on the stabilization/destabilization of different defects which introduce deep and shallow electronic states within the semiconductor bandgap, thus acting on the modulation of the doping and carrier trapping processes. We find a compositional range, for Br < 50%, where halide alloying can be exploited for controlling the dedoping of tin-based perovskites, reaching an optimum also in terms of photoluminescence efficiency. Beyond such a range, carrier trap sites and charge localization phenomena promote nonradiative recombination in the material and limit photovoltaic, and more in general, optoelectronic performance. While these observations suggest that bandgaps above 1.75 eV will need specific passivation strategies to improve the optoelectronic material quality, highly efficient materials are possible at or below a perovskite bandgap of 1.7 eV.

## Supporting Information Available

Experimental section and additional characterization, including SEM images, XRD, steady-state PL, transient absorption spectra, and IPCE curves (PDF)
