# Supplementary material for: How Halide Alloying Influences the Optoelectronic Quality in Tin-Halide Perovskite Solar Absorbers
Source: ACS Energy Lett. 2023 Aug 28;8(9):3876–82. doi: 10.1021/acsenergylett.3c01241 (PMC10496121; doi:10.1021/acsenergylett.3c01241)
Supplement: Supplementary file 1 — nz3c01241_si_001.pdf [file nz3c01241_si_001.pdf]

Supporting Information for

How Halides Alloying Influences the Optoelectronic Quality in Tin-Halide Perovskite Solar Absorbers

Felix J. Berger[1], Isabella Poli[1], Ece Aktas[2], Samuele Martani[1,3], Daniele Meggiolaro,[4] Luca Gregori,[5] , Munirah D. Albaqami[8] Antonio Abate [2], Filippo De Angelis [4,5,6] and Annamaria Petrozza[1,7]*

[1] Center for Nano Science and Technology @PoliMi, Istituto Italiano di Tecnologia, via Rubattino 81, 20134, Milano, Italy.

[2] Department of Chemical, Materials and Production Engineering, University of Naples Federico II; Piazzale Vincenzo Tecchio, 80, 80125 Napoli, Italy.

[3] Physics Department, Politecnico di Milano, Piazza L. da Vinci, 32, 20133 Milano, Italy.

[4] Computational Laboratory for Hybrid/Organic Photovoltaics (CLHYO), Istituto CNR di Scienze e Tecnologie Chimiche 'Giulio Natta' (CNR-SCITEC), 06123, Perugia, Italy.

[5] Department of Chemistry, Biology and Biotechnology, University of Perugia, 06123, Perugia, Italy.

[6] SKKU Institute of Energy Science and Technology (SIEST) Sungkyunkwan University, Suwon, Korea 440-746.

[7] Chemistry Department, College of Science, King Saud University, Riyadh 11451, Saudi Arabia

Materials

N,N-Dimethylformamide (DMF, anhydrous, 99.8%), dimethylsulfoxide (DMSO, anhydrous, 99.9%) anisole (anhydrous, 99.7%) were purchased from Sigma-Aldrich; tin(II) iodide (SnI_2, 99.99%) and tin(II) fluoride (SnF_2, 99%) were purchased from Sigma-Aldrich. SnBr2 was purchased by TCI. All chemicals were used without any further purification. Glass substrates were cleaned in acetone and isopropyl alcohol (IPA)for 10 min by sonication. The cleaned glass substrates were treated with oxygen plasma for 10 min before any further deposition. Thin-film perovskite deposition was done in a N_2-filled glovebox and thin-films were glass encapsulated immediately after thermal annealing (in the glovebox).

To make $MASn(I_{1-x}Br_x)_3$ **thin films**, the precursor solution was obtained by mixing varying ratios of solutions of (i) MAI + SnI_2 (1.2 M) and (ii) MAI + $SnBr_2$ (1.2 M). $MASnBr_3$ precursor solution was prepared by mixing MABr + $SnBr_2$ (1.2M). To make $FASn(I_{1-x}Br_x)_3$ thin-films, the precursor solution was obtained by mixing varying ratios of equimolar solutions of (i) FAI + SnI_2 (1.2 M) and (ii) FAI + $SnBr_2$ (1.2 M). $FASnBr_3$ precursor solution was prepared by mixing FABr + $SnBr_2$ (1.2M). All solutions were prepared using mixed solvents DMF:DMSO (4:1), stirred at room temperature for 30 min and then filtered through 0.20-µm PTFE membrane before use. The perovskite films were deposited with one-step spin-coating procedures at 4000 r.p.m. for 50 s. Anisole (80 µl) was dropped on the spinning substrate 25 s before the end of the procedure. The substrates were annealed at 100°C for 30 min. When specified, 10

mol% SnF_2 with respect to the SnI_2+$SnBr_2$ content was added to the precursor solution. Thin films were prepared in the same way as the pristine thin films.

For the preparation of **solar cell devices,** indium tin oxide (ITO) coated glass substrates were sequentially cleaned in Hellmanex 2% deionised (DI) water solution, DI water, acetone, and 2-propanol by sonication at 40 °C for 15 min. The PEDOT-complex solution was prepared by diluting PEDOT (HTL3 from Clevios) with anhydrous toluene (1:4 v:v). ITO substrates were treated by O_2 plasma cleaning for 15 min before depositing the PEDOT layer into a N2-filled glove box. The PEDOT solution was spin coated onto ITO substrates at 4000 rpm for 30 s, and then baked on a hotplate at 150 °C for 10 min. After annealing, the ITO/PEDOT substrates were allowed to cool down to room temperature naturally. Subsequently, a thin layer of Al_2O_3 nanoparticles dispersion was deposited on top of PEDOT layer, as reported in a previous study.[1] Al_2O_3 nanoparticles dispersion (Sigma-Aldrich) was diluted with IPA (1:50 v:v) and spin-coated onto ITO/PEDOT substrates at 4000 rpm for 30 sec and annealed for 5 minutes at 100 °C. $FASn(I_{0.5}Br_{0.5})_3$, $FASn(I_{0.67}Br_{0.33})_3$, $FASn(I_{0.83}Br_{0.17})_3$ and $FASnI_3$ solar cells were prepared by mixing 1.2 M solutions of $FASnI_3$ and $FASnBr_3$ to obtain the desired I/Br ratio. N,N-Diethylformamide (DEF):1,3-Dimethyl-3,4,5,6-tetrahydro-2(1H)-pyrimidinone (DMPU) (6:1, v:v) solvent mixture is utilized.[1] The perovskite precursor (with 10% over-stoichiometry of SnI_2 and 5% 1 M $EDAI_2$) was spun at 500 rpm for 5 s and 4000 rpm for 25 seconds. 100 μL of DEE were dropped on the spinning substrate at 21 s from the start of the deposition process. The film was annealed at 100 °C for 30 minutes. The electron-transport layer was prepared by evaporating a C60 layer (40 nm) and BCP (9 nm) as buffer layer at a 10^{-6} mbar vacuum level with a deposition rate between 0.1 and 0.3 Å/s, respectively. Finally, a 120 nm thick layer of silver was thermally evaporated as the top electrode.

Experimental Methods

SEM images were obtained using a MIRA3 TESCAN microscope with an accelerating voltage of 5 kV. Perovskite films were prepared on ITO substrates.

XRD patterns were recorded with a Bruker D8 Advance diffractometer with Bragg–Brentano geometry equipped with a Cu Ka1(l= 1.544060 Å) anode, operating at 40 kV and 40 mA. All the diffraction patterns were collected at room temperature, with a step size of 0.05 in symmetric scan reflection mode and an acquisition time of 1 s.

UV-vis steady state absorption spectra were measured on perovskite thin films deposited on bare glass using a UV/VIS/NIR spectrophotometer Lambda 1050, PerkinElmer, with a step size of 1 nm.

In **PDS** the absorption spectrum of a thin film can be obtained by monitoring the change of refractive index of the medium surrounding its surface. The sample is submerged in tetradecafluorohexane and illuminated by monochromatic light provided by a SuperK Extreme supercontinuum laser, coupled with a SuperK SELECT acousto-optic tunable filter (NKT Photonics). The thermal relaxation of the photogenerated carriers creates a thermal gradient in the portion of liquid around the sample’s surface. This, in turn establishes a refractive index gradient (mirage effect) that deflects a He-Ne laser (JDSU)) aligned parallel and in close proximity to the sample’s surface. The deflection of the He-Ne laser is measured by a quadrant detector (PDQ80A, Thorlabs). The absorption coefficient, at each wavelength, is proportional to the amplitude of the deflection signal, granting high sensitivity thanks to the scatter-free detection. The excitation light is modulated by a chopper (4 Hz) to enable lock-in detection (SR830), and by changing its wavelength we retrieve the full absorption spectrum (after normalizing for the power spectrum of the laser). Long-pass filters are used to prevent the leakage of straylight.

Photoluminescence measurements were collected under continuous wave excitation (unless specified otherwise) by a 450 nm diode laser. The PL is acquired with a Maya 2000 pro spectrometer from Ocean Optics. The excitation intensity is adjusted depending on the experiment and specified in the text. PL was measured in air on glass encapsulated samples. For relative PLQY measurements, the integrated photoluminescence was measured at varying excitation intensities and plotted as relative PLQY=I_{PL}/I_{pump}.

Absolute PLQY were obtained from measurements performed in an integrating sphere (Labsphere) on encapsulated thin films deposited on non-conductive glass. Excitation was provided by a c.w. diode laser (375 nm) and spectra acquired through an optical fiber coupled from the sphere to a spectrometer (Ocean Optics Maya Pro 2000). PLQY values were calculated employing the method proposed by de Mello et al.[2] using the equations: $PLQY = \frac{P_C-(1-A)P_B}{L_A A}$ and $A = 1 - \frac{L_C}{L_B}$, where P_B and P_C are the integrated intensity of the diffused PL when the sample is placed inside the sphere out of the laser beam path and directly hit by the laser, respectively. L_A, L_B and L_C are the integrated intensity of the excitation light when the sample is out of the sphere, inside the sphere and out of the laser beam path and in the sphere and directly hit by the laser, respectively.

Transient absorption spectroscopy (TA) was collected in transmission geometry. An amplified femtosecond laser (Light Conversion Pharos) generated pulses of ~280 fs centred at 1030 nm. A broadband white light probe is generated by focusing the pulses into a thin sapphire plate. At short delays (<5 ns), the third harmonic of the fundamental provided the pump light (343 nm). At long delays (>1 ns), pump light at 355 nm was provided by the third harmonic of a Q-switched Nd: YVO4 laser (Innolas Picolo), which was electronically triggered and synchronized to the femtosecond laser via an electronic delay generator. The data acquired in the two-time regimes were combined, with a small scaling factor applied to overlap signal amplitudes between 2 and 4 ns. Kinetics are obtained by integrating over a wavelength window of 40 nm centred at the peak of the main photo-bleach (PB) at the band edge.

Electrical conductivity measurements were obtained by depositing the perovskite film onto Au gold stripe contacts and was calculated =l/Rwt, where l is the length of the Au contacts (0.7 cm), R is the average resistance, t is the thickness of the perovskite film and w is the width between the 2 Au contacts (0.5 cm). The resistance R was measured by using a 2-point electrical probe. An Agilent B1500A Semiconductor Device Parameter Analyzer (SPA) was used to impose a voltage sweep from -1 V to 1 V between the two probes and the corresponding values of current were recorded.

When specified, a 4-point electrical probe was used to measure the resistance R by means of a cylindrical four-point probe head (100 µm diameter tips, 1mm spacing and 60g + load) combined with a HM21 Hand Held Meter (jandel engineering ltd).

Hall effect measurements were obtained using a Hall effect measurement system (semiautomatic) (HMS5300, Ecopia) using Van Der Pauw method with constant current source and 0.51 Tesla permanent magnet.

Computational Details

Defect calculations have been carried out in the 2 x 2 x 2 supercells (384 atoms) of the $MASnI_3$, $MASnI_{1.5}Br_{1.5}$, and $MASnBr_3$ systems. Starting from the experimental tetragonal phase of $MASnI_3$ [3] with cell parameters a = b and c respectively of 8.76 and 12.43 Å, iodine atoms were progressively replaced by bromine atoms to build the $MASnI_{1.5}Br_{1.5}$, and $MASnBr_3$ pristine phases. In the procedure, the equilibrium structures of the $MASnI_{1.5}Br_{1.5}$ and $MASnBr_3$ phases have been obtained by fully relaxing the ion positions and the cell parameters with the Quantum Espresso (QE) software package [4] by using the PBE functional [5]

and including DFT-D3 dispersion corrections,[6] using ultrasoft pseudopotentials with a cutoff on the wavefunction of 40 Ryd (320 Ryd on the charge density) and 4x4x2 k-points grids in the Brillouin zone (BZ). For the $MASnI_{1.5}Br_{1.5}$ a symmetric disposition of the Br ion in the octahedra has been used (two Br's in equatorial and one Br in apical position).

Defect calculations in the 2x2x2 supercells were performed with the CP2K software [7], by sampling the BZ at the Γ point. Defect quantities have been calculated by performing geometry optimization with the hybrid PBE0 functional, [8] by including DFT-D3 Van der Waals corrections [6] and by fixing cell parameters at the optimized values found with QE code. In all cases norm-conserving Goedecker-Teter-Hutter pseudopotentials and DZVP Gaussian basis set [9] were used along with a density cutoff of 300 Ryd on the charge density. We used the auxiliary density matrix with the cFIT auxiliary basis set to speed up the hybrid functional calculations.[10,11] Calculated PBE0 band gaps of the pristine phases have been corrected for spin-orbit coupling (SOC) by rigidly applying the SOC shifts in band gaps obtained at the PBE level by using the QE code and the full relativistic form of the ultrasoft pseudopotentials.

Defect formation energies (DFE) have been calculated according to the expressions [12]:

$$DFE\ [X^q] = E[X^q] - E[perf] - \sum_i n_i \mu_i + q(\varepsilon_{VB} + \varepsilon_F) + E^q_{corr} \qquad (1)$$

$$\varepsilon(q/q') = \frac{E[X^q] - E[X^{q'}]}{q' - q} + \frac{E^q_{corr} - E^{q'}_{corr}}{q' - q} - \varepsilon_{VB} \qquad (2)$$

where $E[X^q]$ is the energy of the supercell with defect X in the charge state q; E(perf) is the energy of the perfect (non-defective) supercell; n and μ are, respectively, the number and the chemical potentials of the species added or subtracted to the non-defective system; ε_{VB} and (ε_F) are the valence band energy and the Fermi level. Corrections for image charge interactions E^q_{corr} has been applied by using the Makov-Payne approach (ε = 25). DFEs have been calculated in I/Br medium conditions, as the intermediate chemical potentials between I/Br rich conditions and I/Br poor conditions. The chemical potentials have been set by considering the field of stability of the perovskites delimited by the Sn_{bulk}, SnI_2, SnI_4, $SnBr_2$ and $SnBr_4$ phases. For $MASnI_3$, I-rich conditions $\mu(I) = (\mu(SnI_4) - \mu(SnI_2))/2$, $\mu(Sn) = (2\mu(SnI_2) - \mu(SnI_4))$; I-poor conditions $\mu(I) = (\mu(SnI_2) - \mu(Sn_{bulk}))/2$, $\mu(Sn) = \mu(Sn_{bulk})$. For mixed halide phases, I/Br rich conditions $\mu(Br) = (\mu(SnBr_2)- 2\mu(SnI_2) + \mu(SnI_4))/2$, $\mu(I) = (\mu(SnI_4) - \mu(SnI_2))/2$, $\mu(Sn) = (2\mu(SnI_2) - \mu(SnI_4))$; I/Br poor conditions $\mu(Br) = (\mu(SnBr_2) - \mu(Sn_{bulk}))/2$, $\mu(I) = (\mu(SnI_2) - \mu(Sn_{bulk}))/2$, $\mu(Sn) = \mu(Sn_{bulk})$. For $MASnBr_3$, Br-rich conditions $\mu(Br) = (\mu(SnBr_4) - \mu(SnBr_2))/2$, $\mu(Sn) = (2\mu(SnBr_2) - \mu(SnBr_4))$; Br-poor conditions $\mu(Br) = (\mu(SnBr_2) - \mu(Sn_{bulk}))/2$, $\mu(Sn) = \mu(Sn_{bulk})$.

Supporting Figures

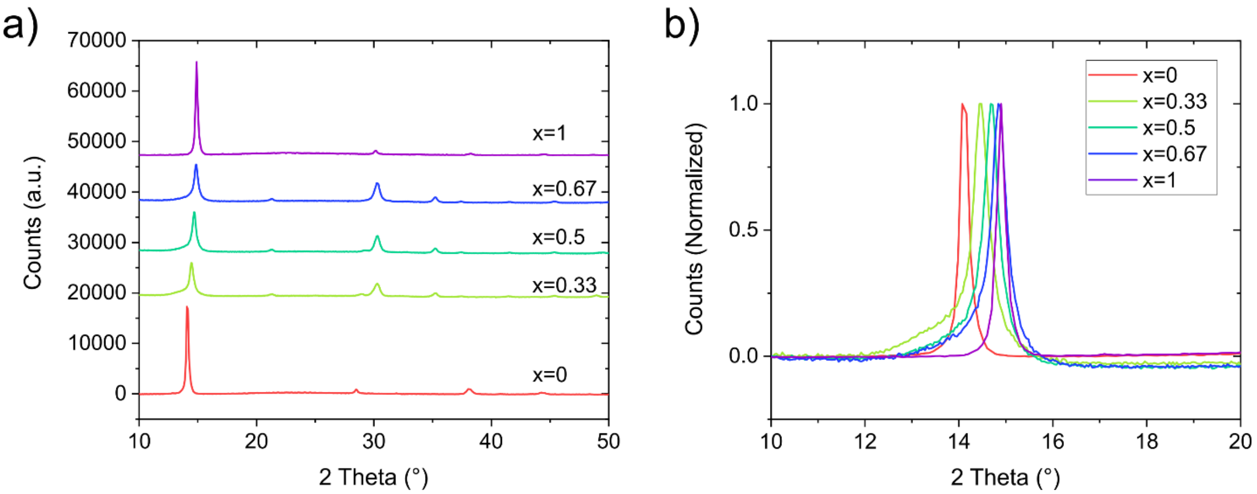

Figure S1 | a) XRD pattern of $MASn(Br_xI_{1-x})_3$ with x=0, 0.17, 0.33, 0.5, 0.67 and 1. b) Magnification of the (110) XRD peak, showing a gradual shift to higher diffraction angles with Br content.

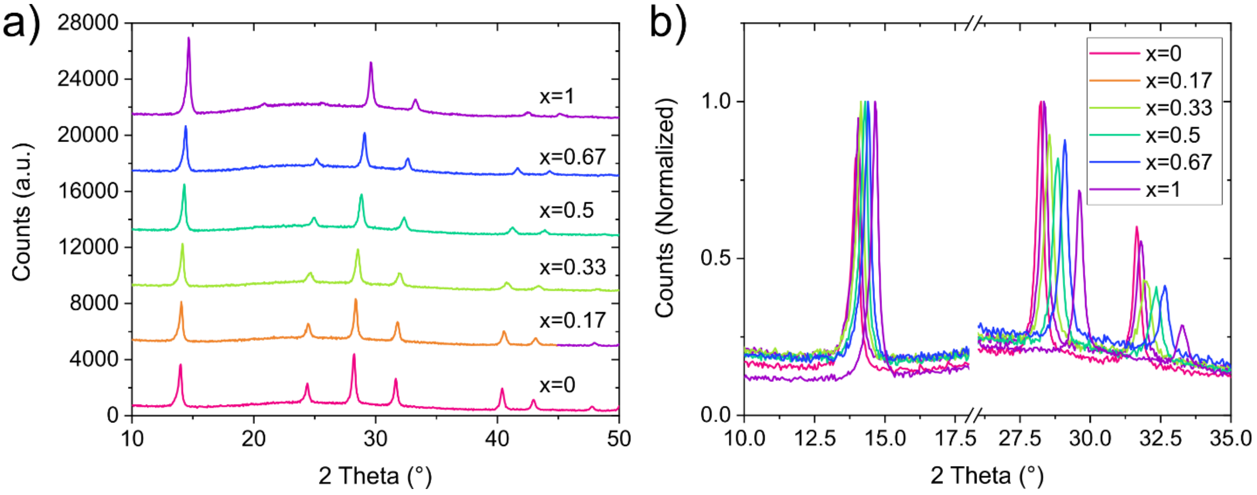

Figure S2| a) XRD pattern of $FASn(Br_xI_{1-x})_3$ with x=0, 0.17, 0.33, 0.5, 0.67 and 1. b) Magnification of the (110) and (220) XRD peak, showing a gradual shift to higher diffraction angles with Br content.

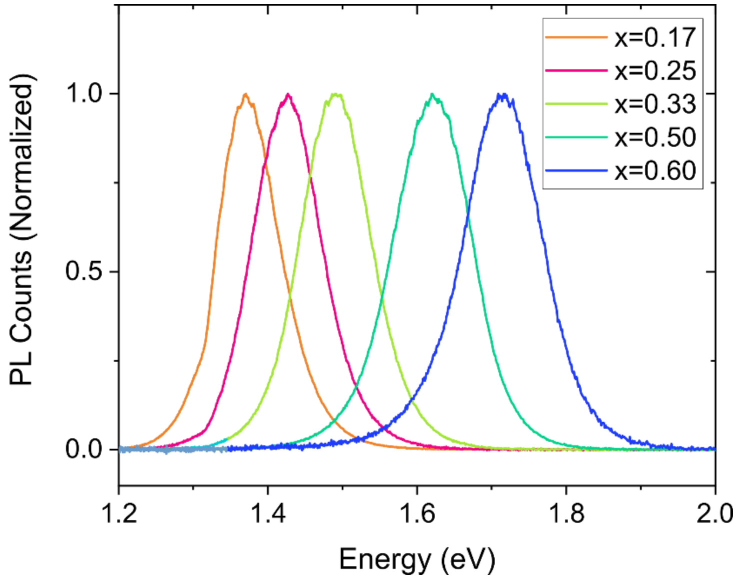

Figure S3 | Photoluminescence spectra of $MASn(I_{1-x}Br_x)_3$ thin films with x=0.17, 0.25, 0.33, 0.5 and 0.6.

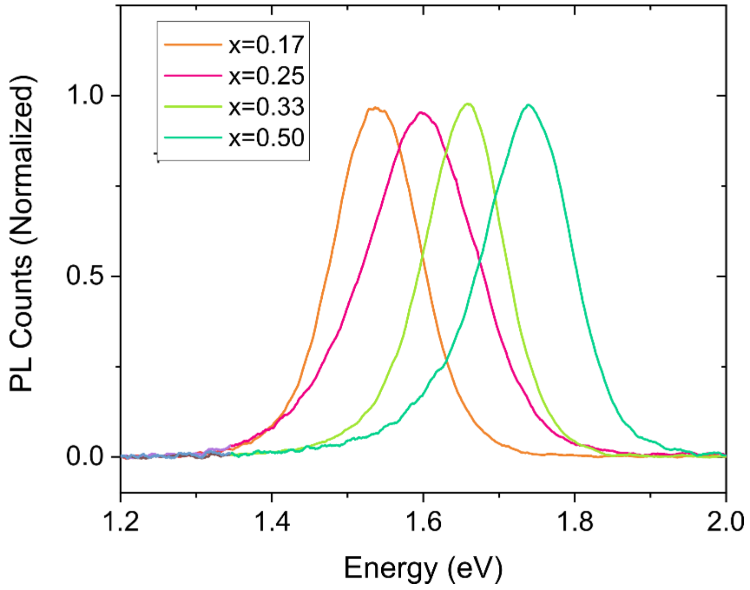

Figure S4 | Photoluminescence spectra of $FASn(I_{1-x}Br_x)_3$ thin films with x=0.17, 0.25, 0.33 and 0.5.

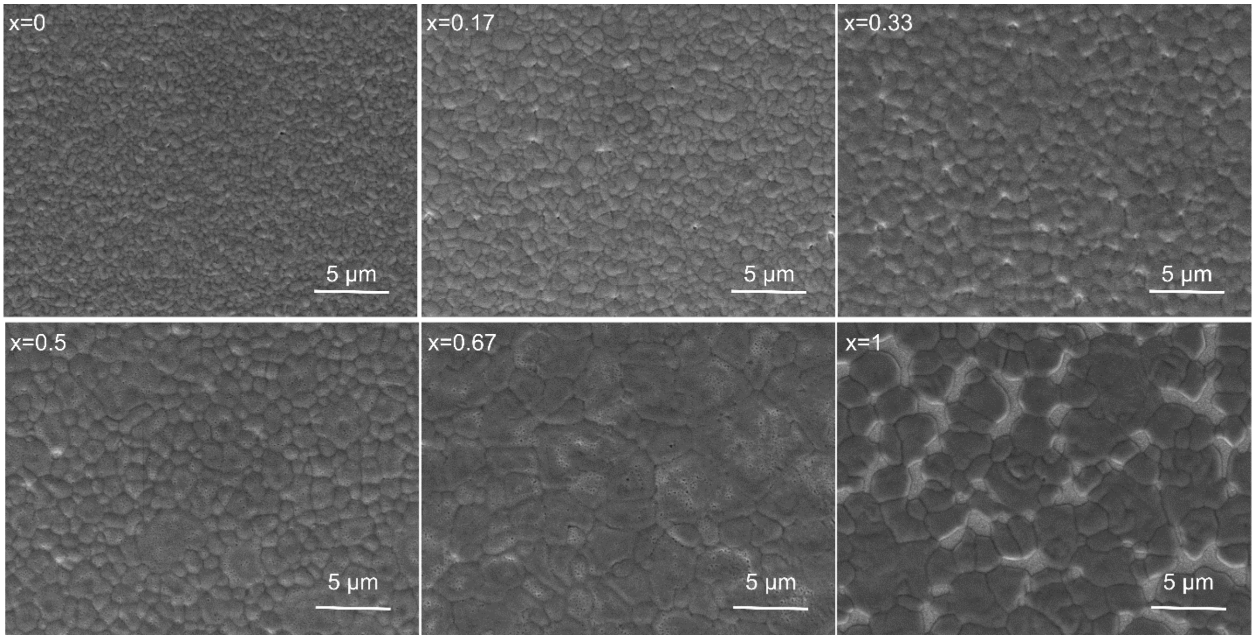

Figure S5 | Top-view SEM images of $FASn(I_{1-x}Br_x)_3$ thin films with x=0, 0.17, 0.33, 0.5, 0.67 and 1.

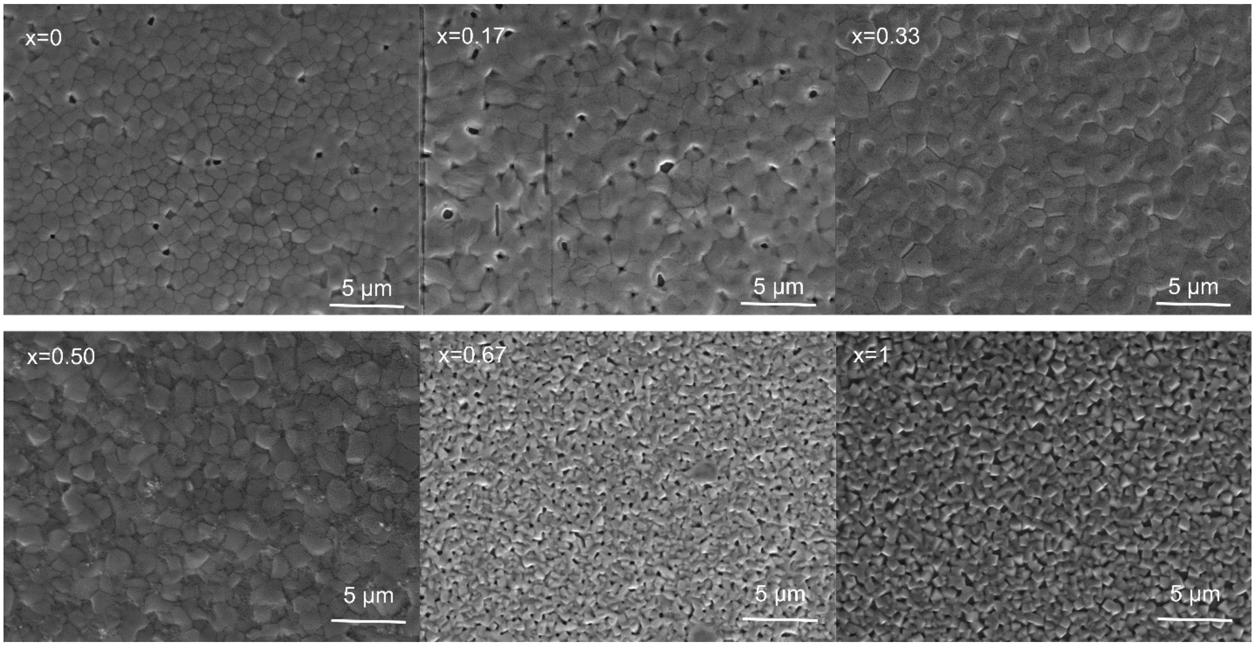

Figure S6 | Top-view SEM images of $MASn(I_{1-x}Br_x)_3$ thin films with x=0, 0.17, 0.33, 0.5, 0.67 and 1.

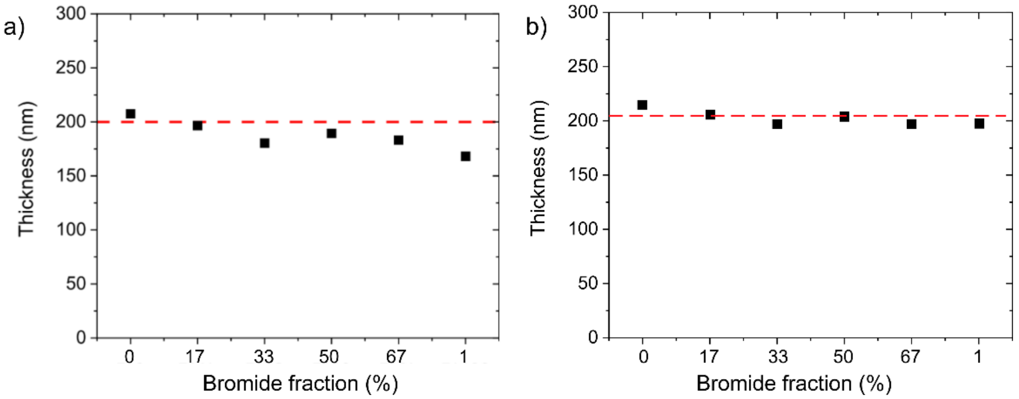

Figure S7 | Thickness of a) $MASn(I_{1-x}Br_x)_3$ and b) $FASn(I_{1-x}Br_x)_3$ thin films with x=0, 0.17, 0.33, 0.5, 0.67 and 1. Thickness was measured using a Dektak profilometer.

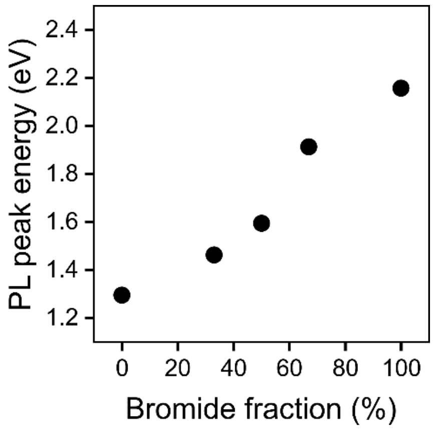

Figure S8 | PL peak position of $MASn(I_{1-x}Br_x)_3$ thin films with increasing bromine fraction (x).

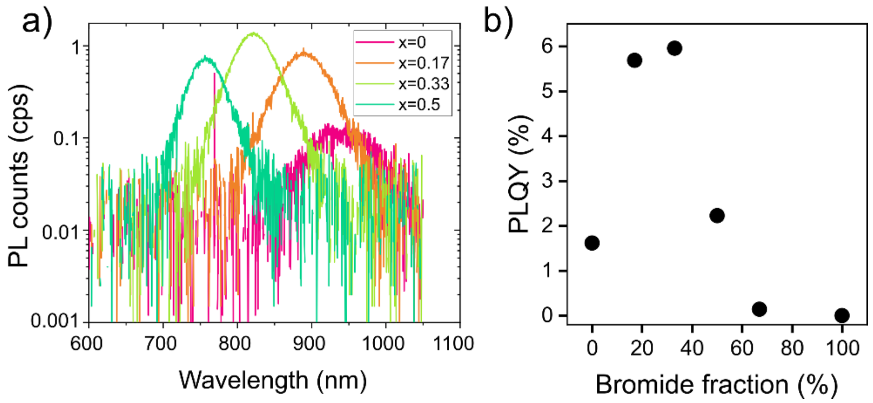

Figure S9 | (a) PL spectra and (b) absolute PLQY values of $MASn(I_{1-x}Br_x)_3$ thin films with increasing bromine fraction (x) measured in an integrating sphere. The PL spectra of compositions with x=0.67 and x=1 are not showed in panel (a) due to the very low intensity.

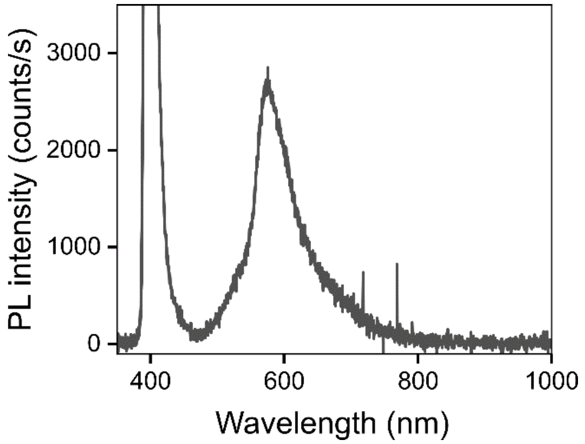

Figure S10 | PL emission of a $MASnBr_3$ thin film with high fluence (excitation of 375 nm, 5mW focused on about 50 µm spot, 1 sec integration time).

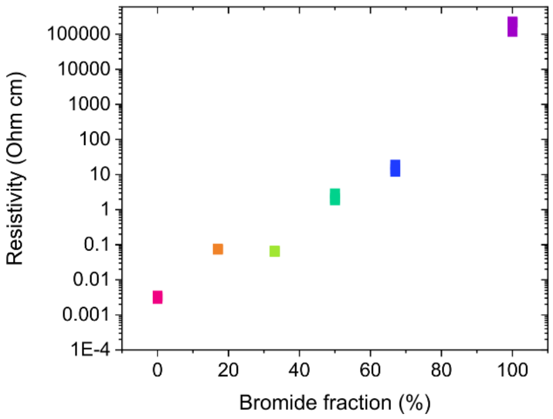

Figure S11 | DC resistivity of $MASn(Br_xI_{1-x})_3$ thin films measured.

Table S1 | Hall effect parameters of $MASn(I_{1-x}Br_x)_3$ perovskite thin films

**Bromide fraction x**	**0**	**0.17**	**0.33**
**Resistivity (Ohm cm)**	6.3×10^{-2}	2.1×10^{-1}	1.4
**Mobility ($cm^2 V^{-1}s^{-1}$)**	2.8	1.59	0.6
**Carrier density (cm^{-3})**	4×10^{19}	1.9×10^{19}	9×10^{18}

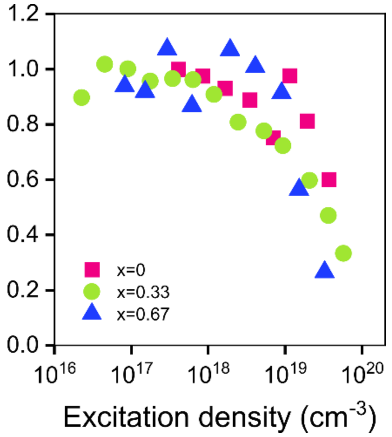

Figure S12 | Relative PLQY measured at different excitation densities using a pulsed excitation centered at 400 nm, with 80 MHz repetition rate.

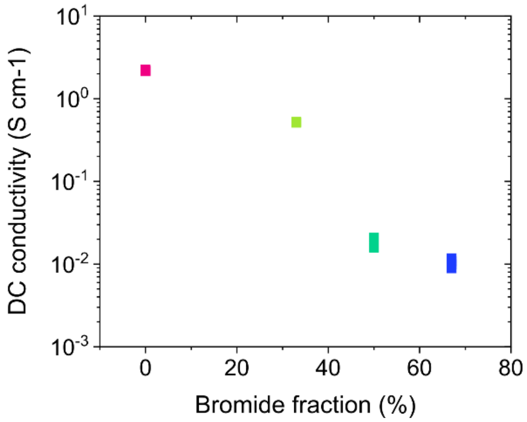

Figure S13 | Conductivity of $MASn(I_{1-x}Br_x)_3$ with x= 0, 0.33, 0.5 and 0.67 fabricated with addition of 10mol% of SnF_2 with increasing Br fractions.

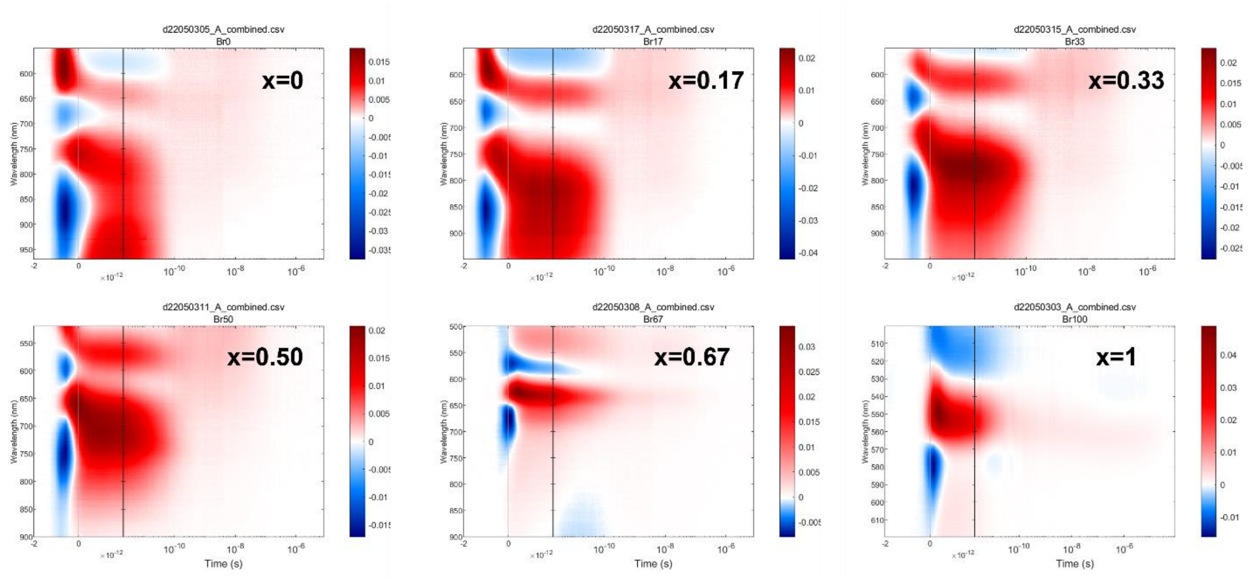

Figure S14 | Two-dimensional transient absorption maps of the $MASn(I_{1-x}Br_x)_3$ perovskite thin films with x=0, x=0.17, x=0.33, x=0.50, x=0.67 and x=1, obtained with a pump of 343 nm / 355 nm and an excitation density = 3 x 10^{19} cm^{-3}

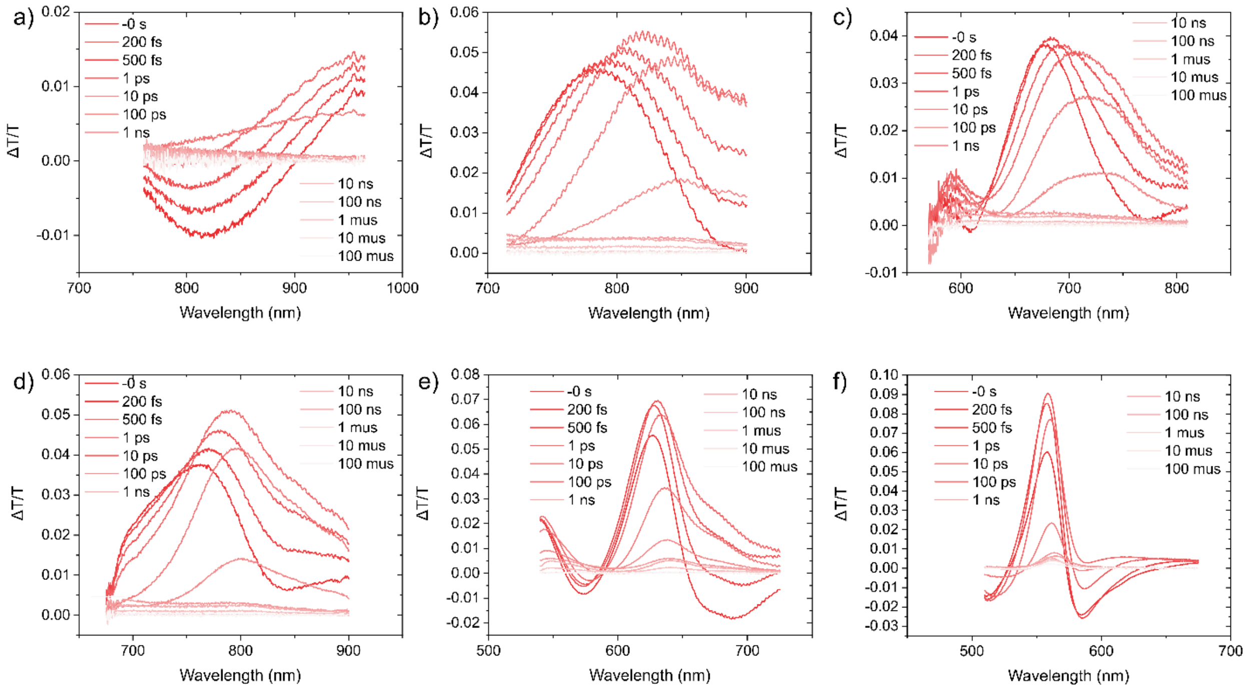

Figure S15 | Representative transient spectra of the main photobleach of photoexcited $MASn(I_{1-x}Br_x)_3$ perovskite thin films with a) x=0 , b) x=0.17 , c) x=0.33 , d) x=0.50 , e) x=0.67 and f) x=1 at different delay times, which are indicated in the legends.

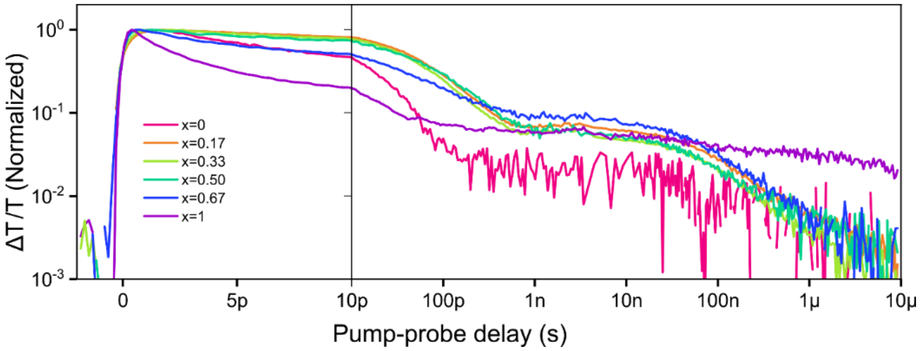

Figure S16 | Normalized kinetics at the bandedge photobleach obtained from transient absorption spectroscopy of $MASn(I_{1-x}Br_x)_3$, with x=0, 0.17, 0.33, 0.50, 0.67 and 1.

Table S2 | Band gaps of the simulated $MASn(I_{1-x}Br_x)_3$ systems, calculated at the PBE0 level of theory without SOC and by rigidly including SOC, as calculated at the PBE level. Calculated DFE@VBM of the most stable acceptor (V_{Sn}^{2-}, I_i^-, Br_i^-) and donor (V_I^+, V_{Br}^+) defects in the different systems at the PBE0 level of theory. All values are in eV.

**Systems**	**Eg (PBE0)**	**Eg (PBE0-SOC)**	**DFE@VBM (V_{Sn}^{2-})**	**DFE@VBM (I_i^- / Br_i^-)**	**DFE@VBM (V_I^+ / V_{Br}^+)**
**$MASnI_3$**	1.62	1.37	0.37	0.42 / -	0.70 / -
**$MASnI_2Br$**	1.88	1.70	0.86	0.72 / 0.61	0.33 / 0.64
**$MASnI_{1.5}Br_{1.5}$**	2.07	1.95	1.27	0.70 / 0.54	0.24 / 0.24
**$MASnBr_3$**	2.37	2.14	1.33	- / 1.01	- / 0.44

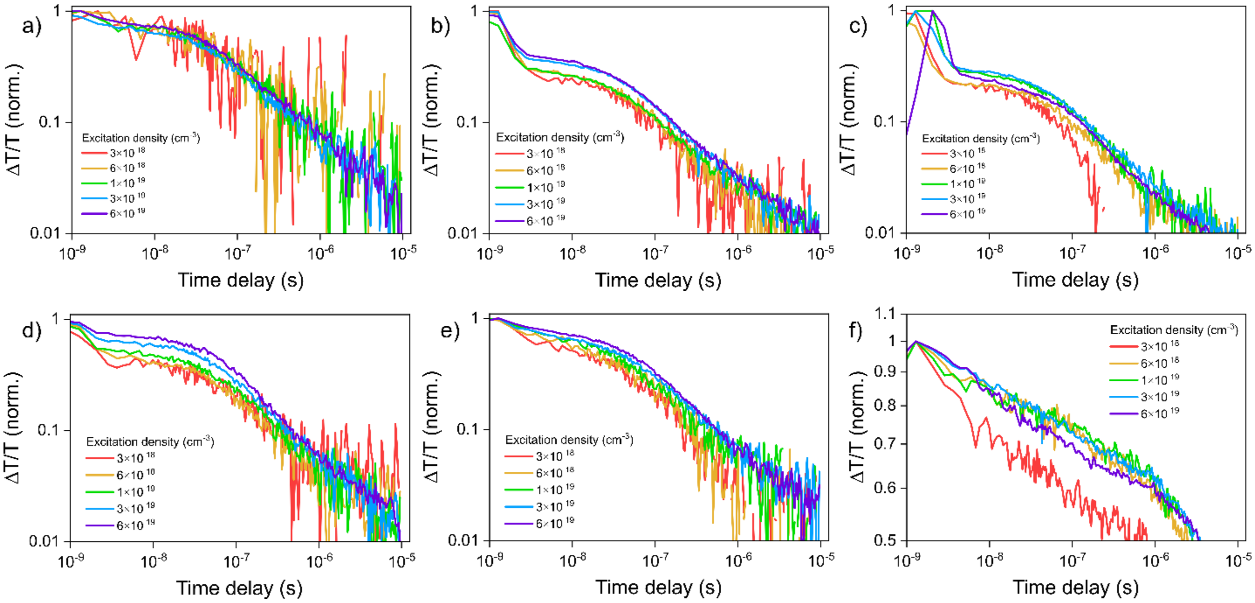

Figure S17 | Normalized kinetics at the bandedge photobleach obtained from transient absorption spectroscopy of $MASn(I_{1-x}Br_x)_3$, with a) x=0 , b) x=0.17 , c) x=0.33 , d) x=0.50 , e) x=0.67 and f) x=1 measured at different excitation densities (indicated in the legends).

Table S3 | Photovoltaic parameters of $FASn(I_{1-x}Br_x)_3$ solar cells with x=0, 0.17, 0.33 and 0.5.

Br fraction (%)	V_{OC} (mV)	Integrated J_{SC} (mA/cm^2)	J_{SC} (mA/cm^2)	FF (%)	PCE (%)
0	536	19.8	20.2	69	7.5
17	580	15.7	16.7	72	6.9
33	669	11.2	11.4	71	5.4
50	469	6.1	4.7	59	1.3

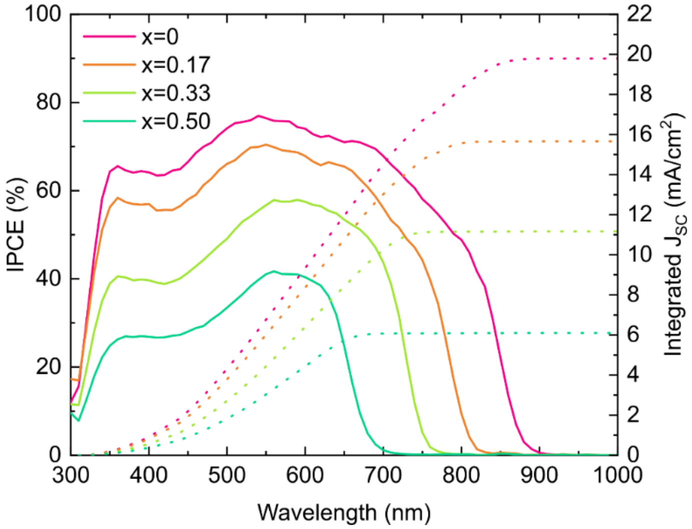

Figure S18 | Incident Photon to Current Efficiency and integrated current of $FASn(I_{1-x}Br_x)_3$ solar cells with x=0, 0.17, 0.33 and 0.5.

References

(1) Di Girolamo, D.; Aktas, E.; Ponti, C.; Pascual, J.; Li, G.; Li, M.; Nasti, G.; Alharthi, F.; Mura, F.; Abate, A. Enabling Water-Free PEDOT as Hole Selective Layer in Lead-Free Tin Perovskite Solar Cells. *Mater Adv* **2022**, *3* (24), 9083–9089. https://doi.org/10.1039/d2ma00834c.

(2) De Mello, J. C.; Wittman, H. F.; Friend, R. H. An Improved Experimental Determination of External Photoluminescence Quantum Efficiency. *Advanced Materials* **1997**, *9*, 230–232. https://doi.org/10.1002/adma.19970090308.

(3) Stoumpos, C. C.; Malliakas, C. D.; Kanatzidis, M. G. Semiconducting Tin and Lead Iodide Perovskites with Organic Cations: Phase Transitions, High Mobilities, and near-Infrared Photoluminescent Properties. *Inorg Chem* **2013**, *52* (15), 9019–9038. https://doi.org/10.1021/ic401215x.

(4) Giannozzi, P.; Baroni, S.; Bonini, N.; Calandra, M.; Car, R.; Cavazzoni, C.; Ceresoli, D.; Chiarotti, G. L.; Cococcioni, M.; Dabo, I.; Corso, A. D.; de Gironcoli, S.; Fabris, S.; Fratesi, G.; Gebauer, R.; Gerstmann, U.; Gougoussis, C.; Kokalj, A.; Lazzeri, M.; Martin-Samos, L.; Marzari, N.; Mauri, F.; Mazzarello, R.; Paolini, S.; Pasquarello, A.; Paulatto, L.; Sbraccia, C.; Scandolo, S.; Sclauzero, G.; Seitsonen, A. P.; Smogunov, A.; Umari, P.; Wentzcovitch, R. M. QUANTUM ESPRESSO: A Modular and Open-Source Software Project for Quantum of Materials. *Journal of Physics: Condensed Matter* **2009**, *21* (39), 395502. https://doi.org/10.1088/0953-8984/21/39/395502.

(5) Perdew, J. P.; Burke, K.; Ernzerhof, M. Generalized Gradient Approximation Made Simple. *Phys Rev Lett* **1996**, *77* (18), 3865–3868.

(6) Grimme, S.; Antony, J.; Ehrlich, S.; Krieg, H. A Consistent and Accurate Ab Initio Parametrization of Density Functional Dispersion Correction (DFT-D) for the 94 Elements H-Pu. *Journal of Chemical Physics* **2010**, *132* (15). https://doi.org/10.1063/1.3382344.

(7) VandeVondele, J.; Krack, M.; Mohamed, F.; Parrinello, M.; Chassaing, T.; Hutter, J. Quickstep: Fast and Accurate Density Functional Calculations Using a Mixed Gaussian and Plane Waves Approach. *Comput Phys Commun* **2005**, *167* (2), 103–128. https://doi.org/https://doi.org/10.1016/j.cpc.2004.12.014.

(8) Adamo, C.; Barone, V. Toward Reliable Density Functional Methods without Adjustable Parameters: The PBE0 Model. *J Chem Phys* **1999**, *110* (13), 6158–6170. https://doi.org/10.1063/1.478522.

(9) VandeVondele, J.; Hutter, J. Gaussian Basis Sets for Accurate Calculations on Molecular Systems in Gas and Condensed Phases. *J Chem Phys* **2007**, *127* (11), 114105. https://doi.org/10.1063/1.2770708.

(10) Freysoldt, C.; Grabowski, B.; Hickel, T.; Neugebauer, J.; Kresse, G.; Janotti, A.; Van De Walle, C. G. First-Principles Calculations for Point Defects in Solids. *Rev Mod Phys* **2014**, *86* (1), 253–305. https://doi.org/10.1103/RevModPhys.86.253.

(11) Komsa, H. P.; Rantala, T. T.; Pasquarello, A. Finite-Size Supercell Correction Schemes for Charged Defect Calculations. *Phys Rev B Condens Matter Mater Phys* **2012**, *86* (4). https://doi.org/10.1103/PhysRevB.86.045112.

(12) Zhang, S. B.; Northrup, J. E. Chemical Potential Dependence of Defect Formation Energies in GaAs: Application to Ga Self-Diffusion. *Phys Rev Lett* **1991**, *67*, 17.

(13) Meggiolaro, D.; Ricciarelli, D.; Alasmari, A. A.; Alasmary, F. A. S.; Angelis, F. De. Tin versus Lead Redox Chemistry Modulates Charge Trapping and Self Doping in Tin/Lead-Iodide Perovskites. *J. Phys. Chem Lett.* **2020**, *11*, 3546–3556. https://doi.org/10.1021/acs.jpclett.0c00725.
